# Association between Sex-Related *ALOX5* Gene Polymorphisms and Lung Atopy Risk

**DOI:** 10.3390/jcm12082775

**Published:** 2023-04-08

**Authors:** Davida Mirra, Renata Esposito, Giuseppe Spaziano, Concetta Rafaniello, Pasquale Iovino, Erika Cione, Luca Gallelli, Bruno D’Agostino

**Affiliations:** 1 Department of Environmental Biological and Pharmaceutical Sciences and Technologies, University of Campania “Luigi Vanvitelli”, 81100 Caserta, Italy; 2 Campania Regional Centre for Pharmacovigilance and Pharmacoepidemiology, 80138 Naples, Italy; 3 Department of Experimental Medicine, Section of Pharmacology “L. Donatelli”, University of Campania “Luigi Vanvitelli”, 80138 Naples, Italy; 4 Department of Pharmacy, Health and Nutritional Sciences, University of Calabria, 87036 Rende, Italy; 5 Clinical Pharmacology and Pharmacovigilance Unit, Department of Health Sciences, Mater Domini Hospital, University of Catanzaro, 88100 Catanzaro, Italy

**Keywords:** *ALOX5*, single nucleotide polymorphisms, leukotrienes, gender differences, lung atopy

## Abstract

Atopy is an exaggerated IgE-mediated immune response to foreign antigens in which metabolic abnormalities of the leukotrienes (LTs) pathway play a crucial role. Recent studies have described sex as a key variable in LT biosynthesis, partly explaining why treatment with anti-LT drugs in atopic subjects leads to better control of symptoms in women. In addition, variability in LT production is often associated with single nucleotide polymorphisms (SNPs) in the arachidonate 5-lipoxygenase (*ALOX5*) gene, which encodes the leukotriene-synthesizing enzyme machinery, 5-lipoxygenase (*5-LO*). This study aimed to investigate whether two SNPs of *ALOX5* are implicated in sex differences in allergic diseases in a prospective cohort of 150 age- and sex-matched atopic and healthy subjects. Rs2029253 and rs2115819 were genotyped using allele-specific RT-PCR, and serum levels of *5-LO* and *LTB4* were measured by ELISA. Both polymorphisms are significantly more common in women than in men, and their influences on LT production vary as a function of sex, leading to a decrease in men’s and an increase in women’s serum levels of *5-LO* and *LTB4*. These data represent a new resource for understanding sex-related differences in lung inflammatory diseases, partly explaining why women are more likely to develop allergic disorders than men.

## 1. Introduction

Pharmacogenomics have become essential in the study of allergic respiratory diseases, as they show how polymorphisms can affect lung function [1,2,3]. Single nucleotide polymorphisms (SNPs) of the leukotrienes (LTs) pathway represent a risk factor for allergic diseases, influencing the severity of symptoms, the course of disease, and the response to drug treatment [4,5]. Indeed, arachidonate 5-lipoxygenase (*ALOX5*) gene, encoding for the leukotriene-synthesizing enzyme machinery 5-lipoxygenase (*5-LO*), is highly polymorphic and often results in the variable production of cysteinyl leukotrienes (CysLTs) and leukotriene B4 (*LTB4*) [6,7,8]. These inflammatory lipid mediators, produced by arachidonic acid oxidative metabolism via multistep enzymatic reactions, show their biological effects by binding to metabotropic receptors (CysLT 1-CysLT2 or BLT1-BLT2) [9,10]. In particular, *LTB4* is a potent chemotactic agent and plays a key role in many inflammatory diseases, including asthma, allergic rhinitis, atopic dermatitis, allergic conjunctivitis, chronic obstructive pulmonary disease, and pulmonary arterial hypertension, which are characterized by dysregulated fatty acid metabolism [11,12,13,14,15,16,17,18]. Understanding the mechanism responsible for this metabolic abnormality has led to the identification of altered expression of enzymes involved in LT biogenesis [19]. Several mutations occur within the *ALOX5* promoter region and are associated with airway hyperresponsiveness and asthma severity [20,21]. Indeed, the promoter region lacks the typical TATA or CCAT boxes but contains eight GC boxes, five of which are arranged in tandem (GGGCGG) and can bind to the SP1 and EGR-1 transcription factors [22]. The Sp1 repeat polymorphism is an important marker for lung inflammatory diseases and consists of the addition of zinc finger (Sp1/Egr-1) binding sites in the region upstream of the ATG translation start site [23,24,25,26]. Moreover, rs2029253 and rs2115819, two intronic variants in the *ALOX5* gene, which consists of an A > G and an A > T substitution in the two alleles, respectively, have been associated with modified transcription activity (altered expression of *ALOX5*) and, consequently, with a different activity of *5-LO* [27,28]. Nevertheless, reported data are still conflicting, with some authors associating them with better responses to anti-LTs and others associating them with reduced responses [29,30]. Montelukast, a cysLTR1 antagonist, is widely used for asthma treatment as an alternative to or in association with Inhaled Corticosteroids (ICS) in steps 3 or 4 of allergic rhinitis treatment, and in the prophylaxis of exercise-induced asthma [31]. Although several papers described the efficacy and safety of montelukast in both asthmatic adults and children, there were significant percentages of non-responders (35 to 78%) [32,33]. In our previous study, we reported that treatment with montelukast in women decreases inflammation, enhances the control of symptoms and management of lung function and results in lower levels of inflammation compared to men [34]. Indeed, recent studies have described sex as a key variable in LT biosynthesis, partly explaining why women are more likely to develop asthma than men and the different responses to anti-LT drugs [35,36]. This variability is often associated with the above-mentioned polymorphisms or with those present in the gene encoding cysLTR1 [30,37,38]. Several studies have reported that polymorphisms in the *ALOX5* gene influence LT production; however, no study has analyzed whether this influence is different between men and women. Therefore, the aim of this study was to assess whether two genetic variations of the *ALOX5* gene, rs2029253 and rs2115819, are implicated in the sex difference in allergic diseases in a well-characterized patient cohort of both atopic and healthy subjects.

## 2. Materials and Methods

### 2.1. Population

Herein, we carried out an observational clinical study on 150 age- and sex-matched subjects with atopy and healthy controls enrolled at the “Mater Domini” Hospital in Catanzaro, Italy. Based on their clinical characteristics, all patients underwent routine venous blood collection in accordance to normal clinical practice. This study belongs to a clinical trial recorded in clinicaltrials.gov (NCT04567212) and was approved by the local Ethics Committee “Calabria Centro”. This work was performed following the Institutional Review Board/Human Subjects Research Committee requirements and the Declaration of Helsinki and the Guidelines for Good Clinical Practice criteria. Prior of the beginning of the study, all patients or legal guardians signed the informed consent.

Inclusion criteria: patients with a diagnosis of atopy in agreement with international guidelines, both sexes, ages between 6 and 85 years.

Exclusion criteria: Subjects with severe asthma or with mixed asthma (asthma and chronic obstructive pulmonary disease) or with active pulmonary infections or immune disorders were not enrolled. In addition, we excluded those who did not sign the informed consent.

### 2.2. Data Collection and Genomic DNA Extraction

Clinical features and treatment data were obtained during enrollment and examined by a team of physicians. EDTA-anticoagulated venous samples were taken after a 12 h overnight fast and analyzed within 2 h of collection. A first centrifugation at 1800× *g* for 10 min at room temperature was performed to gather serum, which was further centrifuged at 1200× *g* for 20 min at 10 °C to completely remove cell debris. Finally, serum samples were aliquoted to prevent freeze–thaw cycles and stored at −80 °C until DNA extraction. Genomic DNA were extracted from serum samples from all participants using a salting-out method [39]. Briefly, SDS (10%) and Proteinase K (1 mg/mL) were added to the samples for cell lysis and protein digestion, and proteins were precipitated using NaCl-saturated distilled water. Finally, the samples were centrifuged, and DNA precipitation was performed using 2-propanol. The extracted DNA were quantified by qubit DNA DS Assay Kit, which ensures simple and accurate quantification of DNA using the Qubit 4 Fluorometer; 1 µL of each sample was quantified.

### 2.3. SNPs Genotyping and Serum 5-LO and LTB4 ELISA Quantification

Target SNPs, rs2029253 (*ALOX5*) and rs2115819 (*ALOX5*) were genotyped via the TaqMan SNP assay (Applied Biosystems, Waltham, MA, USA) and DNA amplification, based on the fluorescence intensity of a fluorogenic probe specific to the target sequence, was assessed by an Applied Biosystems Quanto Studio 5 Real-Time PCR System (Applied Biosystems, Waltham, MA, USA). Thermocycling conditions were as follows: an initial pre-read step at 60 °C for 30 s; a denaturation step at 95 °C for 5 min; 40 cycles of denaturation at 95 °C for 15 s; annealing at 60 °C for 1 min; and a post-read step at 60 °C for 30 s. *5-LO* enzyme and *LTB4* levels were measured in a subgroup of subjects by using ELISA kits and all samples were analyzed in duplicate. Rs2029253 were analyzed in: 4 M (2 healthy and 2 atopic) and 4 W (2 healthy and 2 atopic) without mutation; 4 M (all healthy) and 6 W (3 healthy and 3 atopic) carrying homozygous wild-type allele; 3 M (all healthy) and 4 W (3 healthy and 1 atopic) carrying homozygous variant allele; and 6 M (3 healthy and 3 atopic) and 4 W (2 healthy and 2 atopic) carrying heterozygous alleles. Rs2115819 were analyzed in: 4 M (2 healthy and 2 atopic) and 4 W (2 healthy and 2 atopic) without mutation; 6 M (all healthy) and 8 W (4 healthy and 4 atopic) carrying homozygous wild-type allele; 6 M (all healthy) and 6 W (2 healthy and 4 atopic) carrying homozygous variant allele; and 7 M (4 healthy and 3 atopic) and 6 W (3 healthy and 3 atopic) carrying heterozygous alleles. Briefly, assay samples and buffers were incubated with HRP conjugates in pre-coated plates for one hour. After the incubation, the wells were decanted, rinsed five times and incubated with substrates for the HRP enzyme. The product of the reaction between enzyme and substrate developed a blue complex. Finally, to stop the reaction a stop solution was added, which turned the solution yellow. The intensity of color was measured in a microplate spectrometer reader at 450 nm, and a standard curve was plotted correlating the color intensity (O.D.) to the standards concentration. The *5-LO* and *LTB4* concentrations in each sample were interpolated from this standard curve.

### 2.4. Statistical Analysis

Clinical characteristics and SNP frequency data were expressed as mean ± standard deviation (SD) while *5-LO* and *LTB4* serum levels were expressed as standard error mean (SEM). We used nominal (sex, comorbidity, and treatment) and categorical variables (age or weight) and correlations between clinical characteristics were calculated using the one-way ANOVA followed by the Tukey Multiple Comparison Test. The two-way ANOVA test followed by the Tukey Multiple Comparison Test were chosen to evaluate the differences between the groups. Correlations between serum *5-LO* and *LTB4* levels were calculated using the Spearman correlation test. Statistical analyses were performed using GraphPad software (version 8.0) (GraphPad Software, San Diego, CA, USA). Differences were considered statistically significant at *p* < 0.05.

## 3. Results

### 3.1. Clinical Characteristics

Clinical characteristics and pharmacological therapy of atopic subjects and control participants are described in Table 1.

### 3.2. ALOX5 Mutations Frequencies in Men and Women

*ALOX5* rs2029253 and rs2115819 frequencies were assessed according to sex in both atopic and healthy subjects (Table 2 and Table 3, respectively). In the total population, there were significantly more Women (W) carrying rs2029253 than Men (M) (*p* < 0.01), whereas there was no difference found regarding rs2115819. Moreover, a sub-analysis according to disease status revealed that both atopic and healthy W carrying rs2029253 were significantly more frequent than M (*p* < 0.05 and *p* < 0.01, respectively), as shown in Table 2. A similar trend was reported for rs2115819 in atopic subjects, which was more frequent in W compared to M; while in healthy subjects, M carrying the mutation were significantly more common compared to W (*p* < 0.0001 and *p* < 0.001, respectively), as shown in Table 3.

Both healthy and atopic populations were stratified by allele and genotype into four subgroups for each SNP: (1) total subjects with mutations; (2) subjects with homozygous wild-type allele (A > T in allele 1, the most frequently reported allele in the population); (3) subjects with homozygous variant allele (A > T in allele 2, the least frequently reported allele in the population); and (4) subjects with heterozygous allele (A > T in both alleles). Healthy W with rs2029253 homozygous wild-type and heterozygous alleles were significantly more frequent (*p* < 0.0001, and *p* < 0.001, respectively), whereas the homozygous variant allele was more frequent in M (*p* < 0.0001) (Table 4A). Among atopic subjects, rs2029253 homozygous wild-type and variant allele frequencies were significantly higher in W (*p* < 0.0001, and *p* < 0.0001, respectively), whereas the homozygous variant allele was more frequent in M (*p* < 0.0001) (Table 4B).

The genotype and allele frequency of *ALOX5* rs2115819 showed opposite trends in healthy and atopic populations. Indeed, healthy M carrying rs2115819 homozygous wild-type and variant alleles were significantly more frequent (*p* < 0.001 and *p* < 0.0001, respectively) (Table 5A), whereas the same alleles were more frequent in atopic W (*p* < 0.0001, respectively) (Table 5B). Conversely, heterozygous alleles were more frequent in healthy W and atopic M (*p* < 0.0001, respectively) (Table 5A,B).

### 3.3. 5-LO Serum Levels According to Genotype ALOX5 rs2029253 and rs2115819 Polymorphisms

**Figure 1:**
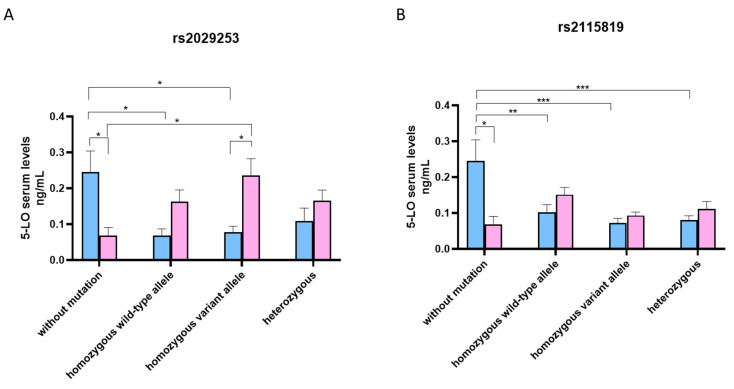
(**A**) Median *5-LO* serum levels in M (blue) and W (pink) according to genotype *ALOX5* rs2029253 polymorphism. Biological replicate: 4 M (2 healthy and 2 atopic) and 4 W (2 healthy and 2 atopic) without mutation; 4 M (all healthy) and 6 W (3 healthy and 3 atopic) carrying homozygous wild-type allele; 3 M (all healthy) and 4 W (3 healthy and 1 atopic) carrying homozygous variant allele; 6 M (3 healthy and 3 atopic) and 4 W (2 healthy and 2 atopic) carrying heterozygous alleles. (**B**) Median 5-LO serum levels in M (blue) and W (pink) according to genotype ALOX5 rs2115819 polymorphism. Biological replicate: 4 M (2 healthy and 2 atopic) and 4 W (2 healthy and 2 atopic) without mutation; 6 M (all healthy) and 8 W (4 healthy and 4 atopic) carrying homozygous wild-type allele; 6 M (all healthy) and 6 W (2 healthy and 4 atopic) carrying homozygous variant allele; 7 M (4 healthy and 3 atopic) 6 W (3 healthy and 3 atopic) carrying heterozygous alleles. All samples were run in duplicate and results were shown as means ± SEM. The statistical tests used in these analyses were the one-way analysis of variance followed by the Tukey Multiple Comparison Test. * *p* < 0.05, ** *p* < 0.01, *** *p* < 0.001.

Serum levels of *5-LO* and *LTB4* were assessed in both M and W, mutated and unmutated, and were stratified by alleles. M without mutations showed higher levels of serum *5-LO* compared to subjects presenting the rs2029253 polymorphism. In particular, M carrying the homozygous wild-type allele had the lowest levels of serum *5-LO* (*p* < 0.05) (Figure 1A). Conversely W carrying the rs2029253 polymorphism, had a higher level of circulating *5-LO*, specifically, those with the homozygous variant allele (*p* < 0.05) (Figure 1A). Like the rs2029253 mutation, M without mutations showed higher levels of serum *5-LO* compared to subjects presenting the rs2115819 polymorphism, particularly compared to those carrying the homozygous variant allele (*p* < 0.001) (Figure 1B). In contrast, there were no significant differences between the serum *5-LO* levels of mutated and non-mutated W (Figure 1B).

### 3.4. LTB4 Serum Levels According to Genotype ALOX5 rs2029253 and rs2115819 Polymorphisms

**Figure 2:**
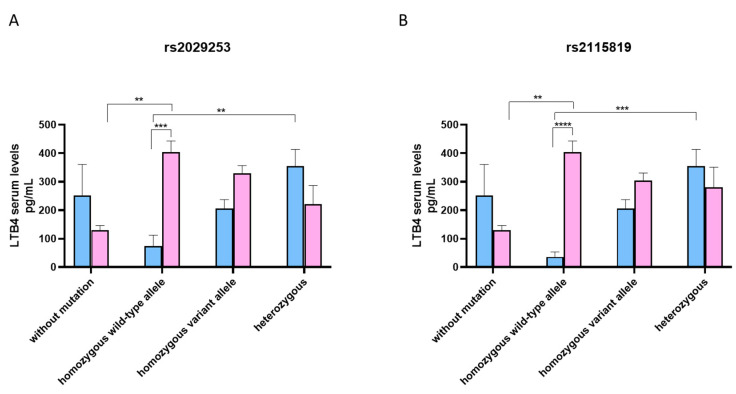
(**A**) Median *LTB4* serum levels in M (blue) and W (pink) according to genotype *ALOX5* rs2029253 polymorphism. Biological replicate: 4 M (2 healthy and 2 atopic) and 4 W (2 healthy and 2 atopic) without mutation; 4 M (all healthy) and 6 W (3 healthy and 3 atopic) carrying homozygous wild-type allele; 3 M (all healthy) and 4 W (3 healthy and 1 atopic) carrying homozygous variant allele; 6 M (3 healthy and 3 atopic) and 4 W (2 healthy and 2 atopic) carrying heterozygous alleles. (**B**) Median 5-LO serum levels in M (blue) and W (pink) according to genotype *ALOX5* rs2115819 polymorphism. Biological replicate: 4 M (2 healthy and 2 atopic) and 4 W (2 healthy and 2 atopic) without mutation; 6 M (all healthy) and 8 W (4 healthy and 4 atopic) carrying homozygous wild-type allele; 6 M (all healthy) and 6 W (2 healthy and 4 atopic) carrying homozygous variant allele; 7 M (4 healthy and 3 atopic) 6 W (3 healthy and 3 atopic) carrying heterozygous alleles. All samples were run in duplicate and results were shown as means ± SEM. The statistical tests used in these analyses were the one-way analysis of variance followed by the Tukey Multiple Comparison Test. ** *p* < 0.01, *** *p* < 0.001, **** *p* < 0.0001.

The serum *LTB4* levels showed a trend similar to the *5-LO* serum levels. Indeed, M without mutations showed higher levels of serum *LTB4* compared to subjects presenting the rs2029253 polymorphism, except for the heterozygous (Figure 2A). Conversely, W with the *ALOX5* rs2029253 polymorphism showed significantly higher levels of *LTB4*, specifically, those carrying the homozygous wild-type allele (*p* < 0.01) (Figure 2A).

M without mutations showed higher levels of serum *LTB4* compared to subjects presenting the rs2115819 polymorphism, except for the heterozygous (Figure 2B). In contrast, W with the *ALOX5* rs2115819 polymorphism showed higher levels of *LTB4*, although it only reached statistical significance in subjects carrying the homozygous wild-type allele (*p* < 0.01) (Figure 2B). 

### 3.5. Correlation between LTB4 and 5-LO Serum Levels

**Figure 3:**
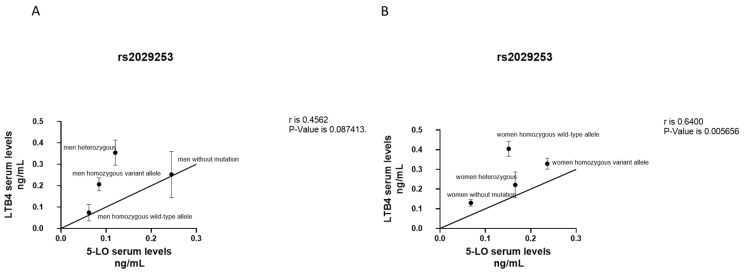
Correlation between *LTB4* and *5-LO* serum levels according to genotype *ALOX5* rs2029253 polymorphism in M (**A**) and W (**B**). Results were shown as means ± SEM. The statistical test used in these analyses was the Pearson correlation test considering 95% confidence interval.

When we correlated *LTB4* and *5-LO* serum levels in M patients with genotype *ALOX5* rs2029253, we did not find a significant association (Figure 3A). Instead, the overall *LTB4* serum levels were strongly directly correlated with circulating *5-LO* levels in W (*p* < 0.01), indicating a stronger association in W carrying homozygous variant allele and homozygous wild type allele (Figure 3B).

**Figure 4:**
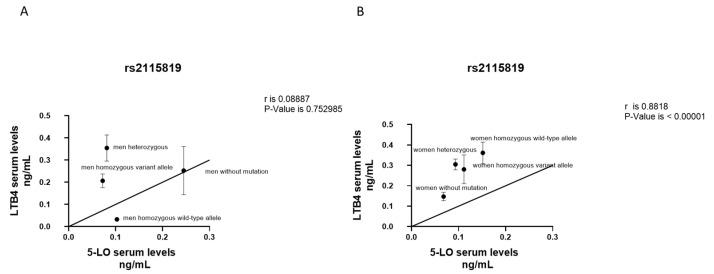
Correlation between *LTB4* and *5-LO* serum levels according to genotype *ALOX5* rs2115819 polymorphism in M (**A**) and W (**B**). Results were shown as means ± SEM. The statistical test used in these analyses was the Pearson correlation test considering 95% confidence interval.

Among M patients, there was no significant association between *LTB4* and *5-LO* serum levels according to the *ALOX5* rs2115819 polymorphism (Figure 4A). Instead, we found a strong and direct correlation in W (*p* < 0.0001), particularly in those carrying the homozygous variant allele and homozygous wild-type allele (Figure 4B).

## 4. Discussion

For the first time, the present study identifies the relationship between *ALOX5* genetic variations and sex-related differences in leukotriene production in a gender-balanced cohort of atopic and healthy subjects. Atopy is an exaggerated IgE-mediated immune response to foreign antigens that makes the immune system more sensitive to common allergic triggers [40]. It is a heterogeneous disease and can be related to other diseases [41,42,43,44,45,46,47,48]. Atopic disorders include various lung inflammatory diseases (e.g., allergic asthma and IgE-mediated components of allergic aspergillosis), allergic rhinitis, conjunctivitis, and atopic dermatitis, in which metabolic abnormalities of the leukotriene pathway play a crucial role [11,12,13,14,15,16,17,18]. The coding gene for the leukotriene-synthesizing enzyme machinery, *ALOX5*, is highly polymorphic and often results in the variable production of CysLTs (*LTC4*, *LTD4*, and *LTE4*) and *LTB4* [6,8]. This variability is often associated with a significantly altered response to montelukast, a cysLTR1 antagonist widely used for the treatment of asthma or allergic rhinitis [30,31,37,38]. Recent studies have described sex as a key variable in LT biosynthesis, partly explaining why women are more likely to develop allergic diseases than men and the differences in response to anti-LT drugs [35,36]. Although several studies have reported that SNPs in *ALOX5* gene could influence LT production, no study has analyzed them in men and woman distinctly. Therefore, we assessed the frequencies of two SNPs in *ALOX5* gene and correlated them with serum *5-LO* and *LTB4* levels in men and women. We showed that *ALOX5* rs2029253 is significantly more common in both atopic and healthy women than in men, while rs2115819 is significantly more common only in atopic W. Furthermore, their influence on LT production varies as a function of gender. Genetic and epigenetic regulation of inflammation are important biological risk factors involved in the development of many diseases, including atopy [49,50]. Rs2029253 and rs2115819 are two intronic variants in the *ALOX5* gene, which consist of an A> G and a A >T substitution in the two alleles, respectively, and have been associated with abnormalities in leukotriene production [27,28]. Nevertheless, reported data are still conflicting, with some authors associating them with better responses to anti-LTs and others associating them with reduced responses. First, Tantsira et al. showed an association between the rs2029253 variant and response to zileuton, but not montelukast. In addition, the same authors reported that *ALOX5* rs2115819 was associated with decrements in response to both zileuton and montelukast therapy [29]. Then, Lima et al. genotyped 28 SNPs in five leukotriene pathway genes associating *ALOX5* rs2115819 with changes in FEV1 and a reduction in exacerbations of asthma in patients treated with montelukast for 6 months [30]. As studies of *ALOX5* polymorphisms have mainly focused on the association with anti-LT drugs, the failure to assess the frequency and effect of these mutations separately between men and women may have resulted in variable data. Our study partly explains this mixture of data in the literature, showing that the genotypes produced from the two SNPs in *ALOX5* gene vary as a function of gender and are more likely associated with the female rather than the male population. In addition to changes in SNP frequency, we also assessed *5-LO* and *LTB4* serum levels directly. Interestingly, despite a different distribution of the two SNPs, they shared a similar influence on LT production, with opposite results in men and women leading to decreased and increased *5-LO* and *LTB4* serum levels, respectively. Moreover, we found that *LTB4* serum levels were strongly and directly correlated with circulating *5-LO* levels only in women, particularly in those carrying homozygous variant allele and homozygous wild type allele. In accordance with studies reporting a higher rate of allergic diseases in woman, our data suggest an overall increase in LT production in the female population carrying these mutations and a higher susceptibility to allergic disorders [51,52]. It is tempting to ascribe the gender differences in the effects of *ALOX5* polymorphisms on LT biosynthesis to the known effects of sex hormones on the expression of genes involved in regulating innate immunity. To date, several clinical and preclinical studies employing animal models of allergic asthma and atopy have described sex differences and the impacts of sex hormones on the of LT biosynthesis in several cells and tissues. Changes in LT production may take place at different levels involving biosynthetic enzyme expression, availability of arachidonic acid, modulation of phosphorylation or subcellular distribution of enzymes [53]. Indeed, there are accumulating evidence indicating that androgens could lower LT levels in vitro and in vivo [54,55,56,57]. Pergola et al. demonstrated that human monocytes derived from females have greater *5-LO* product formation than those derived from males and showed that in vitro stimulation with testosterone metabolites of female-derived monocytes suppressed *5-LO* products biosynthesis [58]. In accordance with this evidence, in our previous study, we showed that montelukast enhances symptom control and management of lung function and decreases inflammation more in women than in men [34]. Therefore, the establishment of the LT pathway and its functions, that could be modulated by genetically controlled differences in the human *ALOX5* gene, may differ between male and female subjects. Previous studies have investigated and reported the association between specific disease-related SNPs and sex. Volf et al. reported that the effects of the 5-HTTLPR polymorphism on brain electrical activity differed as a function of sex, implicating sex-related differences in affective states, emotions, and cognition [59]. In addition, Bi et al. showed that the influence of IL-6 gene polymorphisms on IL-6 biosynthesis and susceptibility to cerebral palsy development is related to sex and gestational age [60]. Therefore, we hypothesize that genetic variation may enhance or weaken the LT-mediated inflammation involved in the pathogenetic process of atopy in a sex-related manner or influence the relationship between genetic factors and the environment.

## 5. Conclusions

To our knowledge, this study gives the first evidence that *5-LO* and *LTB4* participate in the pathogenesis of atopy in a sex-related manner, and that *ALOX5* gene polymorphisms are risk markers for the development of atopy in female patients. Our data, even if preliminary, represent a new resource for understanding sex-related differences in lung inflammatory disease, partly explaining why women are more likely to develop allergic disorders than men. However, we cannot exclude the potential problem of small sample size after genotyping. For this reason, we could not stratify the subjects according to disease status and *5-LO* or *LTB4* levels. Nevertheless, given the low frequencies of these mutations, our findings could be clinically relevant and lead to future studies involving larger populations. The current findings suggest that it may be worthwhile for future research to examine the impacts of gene and sex interactions on lung function. Therefore, additional investigations in larger cohorts are needed to clarify the role of genetic variation in sex variability in anti-LT drug responses and to establish a gender-tailored therapeutic approach for the disease.

## Figures and Tables

**Table 1:** Demographic characteristics and pharmacological treatments of enrolled subjects. Results were shown as means ± SD. The statistical tests used in these analyses were the one-way analysis of variance followed by the Tukey Multiple Comparison Test. NS = not significant.

	Atopic	Healthy	*p* Value
Total participants (N)	36	114	
Mean Age (SD)	39.3(21.8)	59.6(24.5)	NS
Gender (M/F) (N)	18/18	56/58	NS
Ethnicity			
Caucasian	36	114	NS
BMI (SD)	23.2(3.9)	24.5(1.2)	NS
Smoking habit (current/former smoker) (N)	8/3	15/7	<0.05
Comorbidities			
- Hypertension (%) N	5 (13.9%)	16(14%)	NS
- Diabetes N (%)	1 (2.8%)	0	NS
- Other cardiovascular diseases (%)	3 (8.3%)	0	<0.0001
Medications			
- Corticosteroids (%) N	14 (38.9%)	21(18.4%)	<0.0001
- B-blocked (%) N	2 (5.5%)	5(4.4%)	NS
- Ca antagonists (%) N	3 (8.3%)	2(1.7%)	<0.0001 <0.0001
- Antihistamines (%) N	9(25%)	1(0.9%)	<0.0001

**Table 2:** Frequency of mutation in atopic and healthy M and W according to genotype *ALOX5* rs2029253 polymorphism. The statistical tests used in these analyses were the one-way analysis of variance followed by the Tukey Multiple Comparison Test.

rs2029253 *ALOX5*	Men	Women	*p* Value
Tot. with mutation	28/74 (37.9%)	34/76 (44.7%)	<0.01
Atopic with mutation	6/17 (35.3%)	8/19 (42.1%)	<0.05
Healthy with mutation	22/57 (38.6%)	26/57 (45.6%)	0.01

**Table 3:** Frequency of mutation in atopic and healthy M and W according to genotype *ALOX5* rs2115819 polymorphism. The statistical tests used in these analyses were the one-way analysis of variance followed by the Tukey Multiple Comparison Test.

rs2115819 *ALOX5*	Men	Women	*p* Value
Tot. with mutation	34/74 (45.9%)	35/76 (46.1%)	NS
Atopic with mutation	6/17 (35.3%)	13/19 (68.4%)	<0.0001
Healthy with mutation	28/57 (49.1%)	22/57 (38.6%)	<0.001

**Table 4:** Genotype and allele frequency in Healthy (**A**) and Atopic (**B**) M and W, based on the *ALOX5* rs2029253 polymorphism. The statistical tests used in these analyses were the one-way analysis of variance followed by the Tukey Multiple Comparison Test.

**A**			
**rs2029253** **ALOX5**	**Healthy Men**	**Healthy Women**	***p*** **Value**
Without mutation	35/57 (61.4%)	31/57 (54.4%)	<0.001
Homozygous wild-type	7/57 (12.3%)	11/57 (19.3%)	<0.001
Homozygous variant	10/57 (17.5%)	4/57 (7%)	<0.0001
Heterozygous	5/57 (8.8%)	11/57 (19.3%)	<0.0001
**B**			
**rs2029253** ***ALOX5***	**Atopic Men**	**Atopic Women**	***p*** **Value**
Without mutation	11/17 (64.7%)	11/19 (57.9%)	<0.001
Homozygous wild-type	0/17 (0%)	4/19 (21.05%)	<0.0001
Homozygous variant	0/17 (0%)	1/19 (5.3%)	<0.0001
Heterozygous	6/17 (35.3%)	3/19 (15.8%)	<0.0001

**Table 5:** Genotype and allele frequency in Healthy (**A**) and Atopic (**B**) M and W, based on the *ALOX5* rs2115819 polymorphism. The statistical tests used in these analyses were the one-way analysis of variance followed by the Tukey Multiple Comparison Test.

**A**			
**rs2115819** ***ALOX5***	**Healthy Men**	**Healthy Women**	***p*** **Value**
Without mutation	29/57 (50.8%)	35/57 (61.4%)	<0.0001
Homozygous wild-type	9/57 (15.8%)	6/57 (10.5%)	<0.001
Homozygous variant	11/57 (19.3%)	2/57 (3.5%)	<0.0001
Heterozygous	8/57 (14.1%)	14/57 (24.6%)	<0.0001
**B**			
**rs2115819** ***ALOX5***	**Atopic Men**	**Atopic Women**	***p*** **Value**
Without mutation	11/17 (64.7%)	6/19 (31.6%)	<0.0001
Homozygous wild-type	0/17 (0%)	4/19 (21.05%)	<0.0001
Homozygous variant	0/17 (0%)	4/19 (21.05%)	<0.0001
Heterozygous	6/17 (35.3%)	5/19 (26.3%)	<0.0001

## Data Availability

Dr. Spaziano keeps the raw data and figures for each of the experiments performed. The data are available upon request.
